# *In Vivo* Chronic Stimulation Unveils Autoreactive Potential of Wiskott–Aldrich Syndrome Protein-Deficient B Cells

**DOI:** 10.3389/fimmu.2017.00490

**Published:** 2017-05-02

**Authors:** Maria Carmina Castiello, Francesca Pala, Lucia Sereni, Elena Draghici, Donato Inverso, Aisha V. Sauer, Francesca Schena, Elena Fontana, Enrico Radaelli, Paolo Uva, Karla E. Cervantes-Luevano, Federica Benvenuti, Pietro L. Poliani, Matteo Iannacone, Elisabetta Traggiai, Anna Villa, Marita Bosticardo

**Affiliations:** ^1^San Raffaele Telethon Institute for Gene Therapy (SR-Tiget), Division of Regenerative Medicine, Stem Cells and Gene Therapy, IRCCS San Raffaele Scientific Institute, Milan, Italy; ^2^Vita-Salute San Raffaele University, Milan, Italy; ^3^Dynamics of Immune Responses, Division of Immunology, IRCCS San Raffaele Scientific Institute, Milan, Italy; ^4^Laboratory of Immunology and Rheumatic Disease, IGG, Genova, Italy; ^5^Department of Molecular and Translational Medicine, Pathology Unit, University of Brescia, Brescia, Italy; ^6^VIB11 Center for the Biology of Disease, Center for Human Genetics, KU Leuven, Leuven, Belgium; ^7^CRS4, Science and Technology Park Polaris, Pula, Italy; ^8^Cellular Immunology, International Centre for Genetic Engineering and Biotechnology (ICGEB), Trieste, Italy; ^9^Experimental Imaging Center, IRCCS San Raffaele Scientific Institute, Milan, Italy; ^10^Milan Unit, Istituto di Ricerca Genetica e Biomedica, Consiglio Nazionale delle Ricerche, Milan, Italy

**Keywords:** Wiskott–Aldrich syndrome, autoimmunity, B cells, toll-like receptors, apoptotic cells, lymphocytic choriomeningitis virus

## Abstract

Wiskott–Aldrich syndrome (WAS) is a primary immunodeficiency caused by mutations in the gene encoding the hematopoietic-specific WAS protein (WASp). WAS is frequently associated with autoimmunity, indicating a critical role of WASp in maintenance of tolerance. The role of B cells in the induction of autoreactive immune responses in WAS has been investigated in several settings, but the mechanisms leading to the development of autoimmune manifestations have been difficult to evaluate in the mouse models of the disease that do not spontaneously develop autoimmunity. We performed an extensive characterization of *Was*^−^*^**/**^*^−^ mice that provided evidence of the potential alteration in B cell selection, because of the presence of autoantibodies against double-stranded DNA, platelets, and tissue antigens. To uncover the mechanisms leading to the activation of the potentially autoreactive B cells in *Was*^−^*^**/**^*^−^ mice, we performed *in vivo* chronic stimulations with toll-like receptors agonists (LPS and CpG) and apoptotic cells or infection with lymphocytic choriomeningitis virus. All treatments led to increased production of autoantibodies, increased proteinuria, and kidney tissue damage in *Was*^−^*^**/**^*^−^ mice. These findings demonstrate that a lower clearance of pathogens and/or self-antigens and the resulting chronic inflammatory state could cause B cell tolerance breakdown leading to autoimmunity in WAS.

## Introduction

Wiskott–Aldrich syndrome (WAS) is a complex and severe X-linked disorder characterized by microthrombocytopenia, eczema, immunodeficiency, and increased risk to develop autoimmunity and lymphomas, affecting 1–10 out of a million male newborns (1). The protein encoded by the *WAS* gene [WAS protein (WASp)] is a hematopoietic-specific regulator of actin nucleation in response to signals arising at the cell membrane (2, 3). WAS-associated autoimmune complications are frequently observed and can occur also after hematopoietic stem cell transplantation (4). The high incidence of autoimmunity in WAS patients indicates a critical role of WASp in the maintenance of central and peripheral tolerance. Indeed, defective function and/or number of natural T regulatory cells and induced T regulatory cells have been shown in WAS patients and in the mouse model by ours and other groups (5–9). However, several recent evidences suggest a role of B cells in the development of autoimmune manifestations in WAS patients. Earlier reports identified B cell anomalies as mainly due to the defective cytoskeletal-dependent processes resulting in decreased migratory ability, adhesion, and homing (10, 11). These defects may be responsible for the inability of WAS B cells in reaching the site of infection and get properly activated. In addition, phenotypic perturbations reported in WAS patients, including marked reduction of CD21/CD35 coreceptor expression and increased representation of CD21^low^ B cell subset (12–14), could explain abnormalities in antigen capture and presentation resulting in a defective maintenance of B cell tolerance. Immune B cell dysregulation has indeed been confirmed by the presence of circulating autoantibodies in both WAS patients (14–16) and *Was*^−/−^ mice (6, 17). Moreover, in mouse models in which only B cells lacked WASp, the contribution of intrinsic B cell defects in the pathogenesis of autoimmunity has been demonstrated (18, 19).

However, the break of tolerance leading to autoimmunity onset may not be sufficient to induce disease since the contribution of environmental factors seems to be required, as in the case of systemic lupus erythematous (SLE) patients (20). The contribution of external factors to the development of autoimmunity could indeed be critical for WAS patients and could explain the high variability in the frequency of these manifestations, ranging from 25 to 72%, regardless of WASp expression (15, 21, 22). Incomplete clearance of apoptotic cells, bacterial and viral pathogens, due to the impaired functionality of WASp-deficient macrophages (23), could lead to an increased load of antigens. These antigens could be sensed by immune cells through Toll-like receptors (TLRs), whose activation could result in induction of survival and pro-inflammatory signals (24), and may favor the expansion of autoreactive cells in immune-compromised hosts, ultimately leading to autoimmune manifestations in WAS patients.

In this study, we tested the *in vivo* effect of several chronic stimulations (TLR agonist administrations, apoptotic cell injection, and viral infection) in the *Was*^−/−^ mouse model, to define whether incomplete clearance of apoptotic cells and/or pathogen antigens could contribute to the induction of autoimmune manifestations in WAS.

## Materials and Methods

### Mice

C57BL/6 *Was*^−/−^ mice were kindly provided by Siminovitch (25). Wild-type (wt) and *Rag2*^−/−^/γ*c*^−/−^ mice were purchased from Charles River Laboratories Inc. (Calco, Italy). Mice were housed under specific pathogen-free conditions. We used mice ranging from 8 to 12 weeks old, unless otherwise stated.

### *In Vivo* Challenge with TLR Agonists and Apoptotic Cells

Wt and *Was*^−/−^ mice were treated at 6 weeks of age by intravenous (i.v.) injections of LPS (from *Escherichia coli*, Serotype O55:B5, Alexis) at a dose of 1 µg/g once a week for 4 weeks or by intraperitoneal (i.p.) injections of oligodeoxynucleotide CpG (ODN1826, CpG-DNA TCCATGACGTTCCTGACGTT, TIB MOLBIOL s.r.l., Genova, Italy) 2 µg/g twice a week for 4 weeks. In parallel, wt and *Was*^−/−^ control groups received PBS. All animals were sacrificed 1 week after the last injection. For the *in vivo* challenge with apoptotic cells, syngeneic thymocytes were isolated from thymus of age- and sex-matched wt and *Was*^−/−^ mice. Cells were cultured overnight in RPMI 1640 supplemented with 2 mM glutamine, 100 IU/mL penicillin, 100 µg/mL streptomycin (Invitrogen), and 100 µM dexamethasone (Sigma-Aldrich) at a concentration of 2 × 10^7^ cells/mL. The apoptotic phenotype was evaluated with Annexin V-PE and 7-AAD staining (Becton Dickinson) by flow cytometry before injections. Mice were treated once a week for 4 weeks with 10^7^ apoptotic cells in sterile PBS injected i.v. in the tail vein. Wt and *Was*^−/−^ control groups were injected with 10^7^ live cells. Blood and serum were taken from the tail vein before treatment and 7 weeks after the last injection. Proteinuria was determined at the time of sacrifice.

### Infection with Lymphocytic Choriomeningitis Virus (LCMV)

wt and *Was*^−/−^ mice were infected i.v. with 1 × 10^6^ p.f.u. non-cytopathic LCMV (Armstrong strain). Mice were followed for 1 week and then sacrificed to evaluate tissue and serum viral titers, renal damage (proteinuria), specific CD8^+^ T cell response, and generation of autoantibodies anti-double-stranded DNA (dsDNA). Infectious LCMV in serum and tissues of infected animals was quantified by plaque assay on MC57 cells, as previously described (26). Intracellular IFNγ staining was performed in the presence or absence of GP33 stimulation as described (26). Briefly, 5 × 10^5^ splenocytes were cultured in RPMI medium supplemented with 50 U/mL of IL2 and 1 µL/mL of brefeldin A for 5 h in the presence or absence of the specific LCMV peptide (GP33-41). The cells were stained with monoclonal anti-mouse CD8α antibody (clone 53-6.7; Pharmingen, San Diego, CA, USA) and anti-mouse IFNγ antibody (clone XMG 1.2; Pharmingen) and its isotype control antibody [rat immunoglobulin (Ig) G1]. Samples were acquired on a FACS Canto II system (BD) and analyzed with FlowJo software (Tree Star Inc.).

### ELISA

Levels of IgG isotypes, IgM, IgA, and IgE were measured in cell culture supernatants by multiplex assay [Beadlyte Mouse Immunoglobulin Isotyping kit; Millipore] on a Bio-Plex instrument (BioRad, Richmond, VA, USA). Anti-dsDNA antibodies were evaluated by ELISA assay (27). Briefly, polystyrene plates were coated with poly-l-lysine (Sigma-Aldrich, St. Louis, MO) and DNA from calf thymus (Sigma-Aldrich); after coating with 50 µg/mL polyglutamic acid for 45 min and blocking with PBS 3% BSA, serial dilutions of serum starting from 1:20 were incubated overnight. Anti-dsDNA antibodies were detected with alkaline phosphatase (AP)-conjugated goat anti-mouse IgG antibody (Southern Biotech). The score of positivity was assigned to sera that were positive for dilutions of 1:40 or higher. Mouse sera were analyzed to evaluate the presence of antiplatelet antibodies (28). Briefly, wt platelets were isolated from blood collected with the anticoagulant citrate phosphate dextrose and diluted with the same volume of tyrode buffer 1× (5 mM HEPES, 137 mM NaCl, 2.7 mM KCl, 0.4 mM NaH2PO4, 2.8 mM dextrose, pH 7.4). The platelet-rich plasma was centrifuged, and total proteins were extracted from the pellet. Ten micrograms per milliliter of protein extract was coated on 96-well plates and incubated overnight at 4°C. Diluted serum (1:100 and 1:1,000 in blocking buffer) was added and incubated for 2 h. Positivity was revealed by staining with secondary goat anti-mouse IgG-AP antibody (1:2,000 in blocking buffer) followed by pNPP conversion by AP (Sigma-Aldrich). The assay was read at 405 nm on a 680 microplate reader (BioRad).

### ELISpot Assay

Immunoglobulin-secreting cells were analyzed by ELISpot assays performed in plates with nitrocellulose membrane coated with goat α-IgG, IgA, IgM (Southern Biotech), or DNA from calf thymus (Sigma-Aldrich). After blocking with PBS 1% BSA, serial dilutions of total B cells (from 2.5 × 10^5^ to 0.3 × 10^5^) were added and incubated o.n. at 37°C. Plates were then incubated with isotype-specific secondary antibodies, followed by streptavidin-HRP and finally developed with 3-amino-9-ethylcarbazole (Sigma-Aldrich) as a chromogenic substrate. Plates were scanned and counted using the Automated ELISA-Spot Assay Video Analysis System (AELVIS, Germany) to determine the number of spots/well.

### Immunohistochemistry

Indirect immunohistochemistry was performed on tissue sections from *Rag2*^−/−^/γ*c*^−/−^ mice incubated with serum from wt and *Was*^−/−^ mice (27). For each serum, we analyzed the reactivity to four tissues (thyroid, stomach, small intestine, and salivary gland and pancreas). Autoantibodies were revealed by using a goat anti-mouse IgG-HRP antibody (Molecular Probes; Invitrogen, Carlsbad, CA, USA) and diaminobenzidine (DBA, DAKO) followed by hematoxylin counterstaining (Sigma-Aldrich). Slides were examined on a Zeiss Axioplan2 microscope (Carl Zeiss Microimaging, Thornwood, NY, USA).

### Proliferation Assay

Mononuclear cells isolated from spleens of wt and *Was*^−/−^ mice in FACS buffer (PBS 0.3% BSA 0.1% NaN_3_) were stained with the following antibodies: anti-CD19 (1D3), anti-CD45R/B220 (RA3-6B2), anti-CD21/35 (7G6), and anti-CD23 (B3B4), all from BD Pharmingen (San Diego, CA, USA). Follicular (FO; CD21^lo^CD23^+^) and marginal zone (MZ; CD21^hi^CD23^lo^) B cells were cell sorted at FACS Aria BD instrument with a purity >95% and a viability ≥85%. Sorted MZ and FO B cells were labeled with 1.5 µM CFSE (Invitrogen) for 8 min at RT. After quenching the labeling reaction by adding FCS, cells were washed twice. Labeled cells (10^5^ cells/well) were cultured in 96-well plates with the following stimuli: 1 µg/mL CpG (ODN1826, InvivoGen, San Diego, CA, USA) or 1 µg/mL LPS (Sigma-Aldrich). Cell proliferation was analyzed at day 3 and 6 of culture by flow cytometry.

### Proteinuria and Histological Analysis of Renal Damage

Proteinuria was evaluated using Albustix sticks (Bayer). Proteinuria index was scored as follows: 0 < 30 mg/dL, 1 = 30 mg/dL, 2 = 100 mg/dL, 3 = 300 mg/dL, and 4 ≥ 2,000 mg/dL. Four-micrometer thick sections of formalin-fixed, paraffin-embedded kidneys were cut on a microtome and subjected to routine hematoxylin and eosin staining. Microscopic lesions observed during histopathological examination were classified according to the INHAND system (International Harmonization of Nomenclature and Diagnostic Criteria for Lesions in Rats and Mice), and the scores were assigned as previously described (29). In particular, histopathologic findings included tubular mineralization, tubular degeneration/regeneration, inflammatory cell foci, cystic lesions, hyaline tubular casts, glomerulopathy, and interstitial fibrosis. For each finding, we scored presence and severity as follows: 0, absent; 1, minimal; 2, mild; 3, moderate; 4, severe. The sum of all scores assigned for each histopathologic finding defines the final nephropathy score.

### Autoantibody Array

Screening for a broad panel of IgM and IgG autoantibodies was performed using autoantibody arrays (UT Southwestern Medical Center, Genomic and Microarray Core Facility) as previously described (30). Statistical differences between TLR-induced autoantibody profiles were assessed by Global test (31).

### Cytokine Production

Cytokine production was evaluated on serum samples from wt and *Was*^−/−^ mice before and after administration of LPS, CpG, apoptotic cells, or infection with LCMV, by using the Bio-Plex Pro Mouse Cytokine Th17 Panel A 6-plex (IL6, IL17a, IFNg, IL1b, IL10, TNFa) (Biorad) and following the manufacturer’s instruction. The plate was acquired using the Bio-Plex MAGPIX Multiplex Reader (Biorad).

### Statistical Analyses

All results are expressed as median and SD if not stated otherwise. Statistical significance was assessed using the two-tailed Mann–Whitney test. One-way or two-way analysis of variance tests were applied when specified. *P* values <0.05 were considered significant.

## Results

### Autoantibody Production by B Cells of *Was*^−/−^ Mice

**Figure 1:**
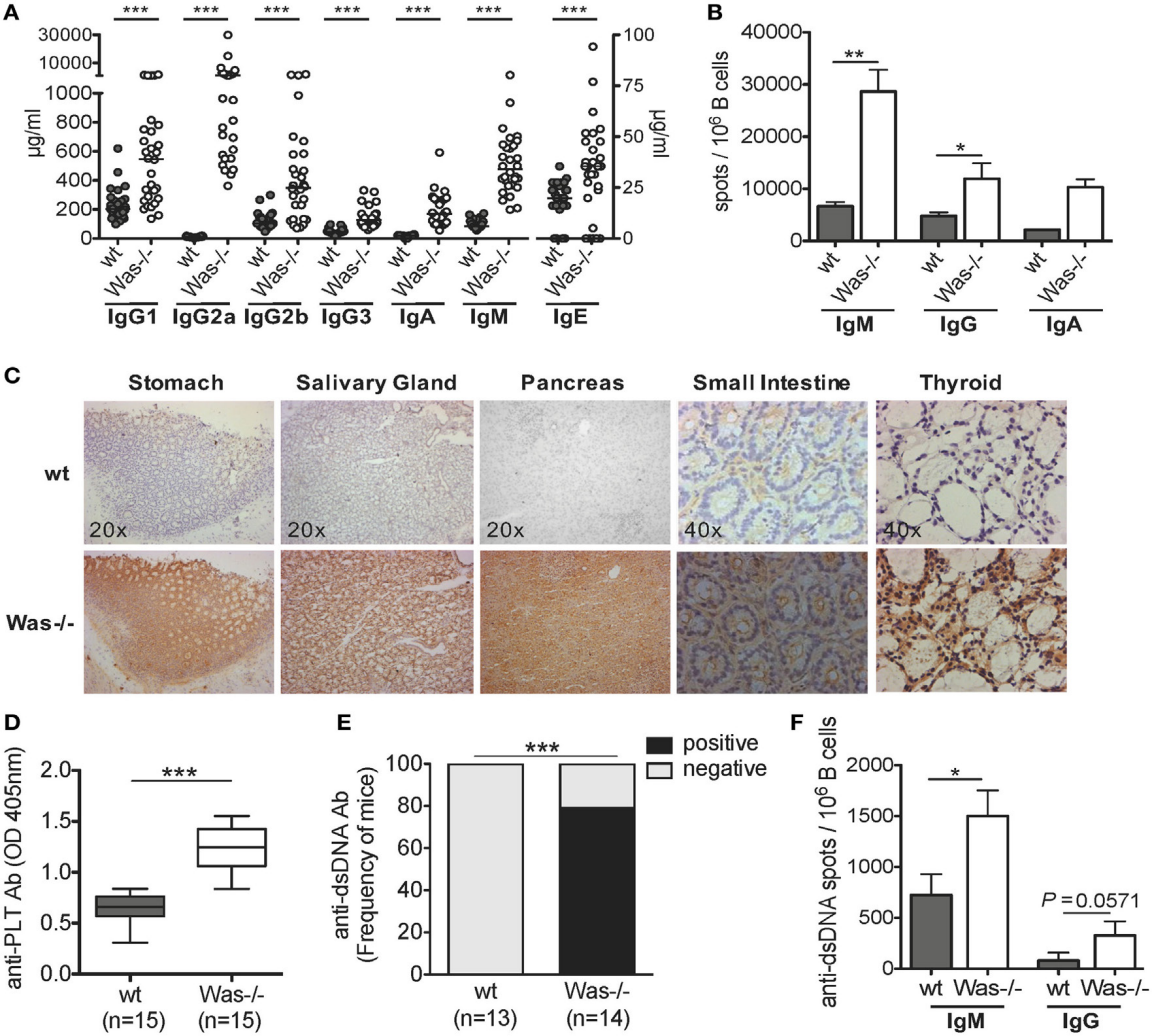
**The presence of autoreactive B cells in *Was^−/−^* mice**. **(A)** Serum levels of immunoglobulins (Igs) subclasses from wild-type wt (*n* = 28) and *Was*^−/−^ (*n* = 30) mice are summarized in the graph. The scale on the right Y axis is specific for IgE levels. Median value is indicated as bar. **(B)** IgM-, IgG-, or IgA-secreting cells were evaluated by ELISpot assay performed on B cells isolated from the spleens of wt and *Was*^−/−^ mice (IgM and IgG: wt *n* = 6, *Was*^−/−^ *n* = 7; IgA: *n* = 3). The mean and SEM are shown. **(C)** Tissue-specific autoantibodies were detected by indirect immunohistochemistry performed on sections derived from different tissues from *Rag2*^−/−^/γ*c*^−/−^ mice incubated with sera of wt (*n* = 6) and *Was*^−/−^ mice (*n* = 6). **(D)** The levels of autoantibodies against platelets present in the sera of wt and *Was*^−/−^ mice were assessed by ELISA and expressed as optical density. Box and whisker plot shows the maximum and minimum value obtained. **(E)** Percentage of mice positive or negative for circulating autoantibodies against double-stranded DNA (dsDNA) evaluated in the sera of wt and *Was*^−/−^ mice and assessed by ELISA. **(F)** Frequency of Ig-secreting cells against dsDNA was assessed by ELISpot assay performed with total splenic wt and *Was*^−/−^ B cells. **(A,B,D,F)** Statistical significant differences were analyzed using the Mann–Whitney test (***P < 0.0001, **P < 0.005, and *P < 0.05). **(E)** Difference was statistically calculated by χ^2^ test (***P < 0.0005).

To address whether the absence of WASp affects the humoral immune response and results in an excessive reactivity against self-antigens, we tested the presence of autoreactive B cells in different settings. Significantly higher concentrations of all classes of total Igs in the sera (Figure 1A) and increased frequency of IgM- and IgG-secreting B cells were found in *Was*^−/−^ mice, compared to wt animals (Figure 1B). We then evaluated the presence of circulating autoantibodies by immunohistochemistry incubating sera of wt and *Was*^−/−^ mice on tissue sections of thyroid, stomach, salivary gland, small intestine, and pancreas derived from Rag2^−/−^/γc^−/−^ mice, to avoid interference of endogenous Igs. We detected a strong immunoreaction in all tissues incubated with sera of *Was*^−/−^ mice as opposed to wt animals (Figure 1C), suggesting the presence of autoreactive Igs targeting different tissues in the sera of mutant mice. Because of the reduced platelet counts in *Was*^−/−^ mice, we evaluated whether the animals could present antiplatelet antibodies in the peripheral blood. As shown in Figure 1D, antiplatelet antibodies were detectable at levels significantly higher in *Was*^−/−^ mice than in age-matched wt mice (8–10 weeks old). The presence of antibodies against dsDNA was also evaluated in wt and *Was*^−/−^ mice by ELISA and ELISpot assays. Seventy-five percent of *Was*^−/−^ mice resulted positive for the presence of anti-dsDNA antibodies, which were completely absent in wt mice (Figure 1E). The increased frequency of B cells producing both IgM or IgG directed against dsDNA in *Was*^−/−^ compared to wt mice was confirmed in ELISpot assays performed on total splenic B cells (Figure 1F).

In conclusion, these data show that *Was*^−/−^ B cells have a higher level of self-reactivity than wt B cells, highlighting a potential breakdown of B cell tolerance in WAS.

### *In Vivo* TLR Ligand Administration Induces Production of Autoantibodies and Tissue Damage in *Was*^−/−^ Mice

**Figure 2:**
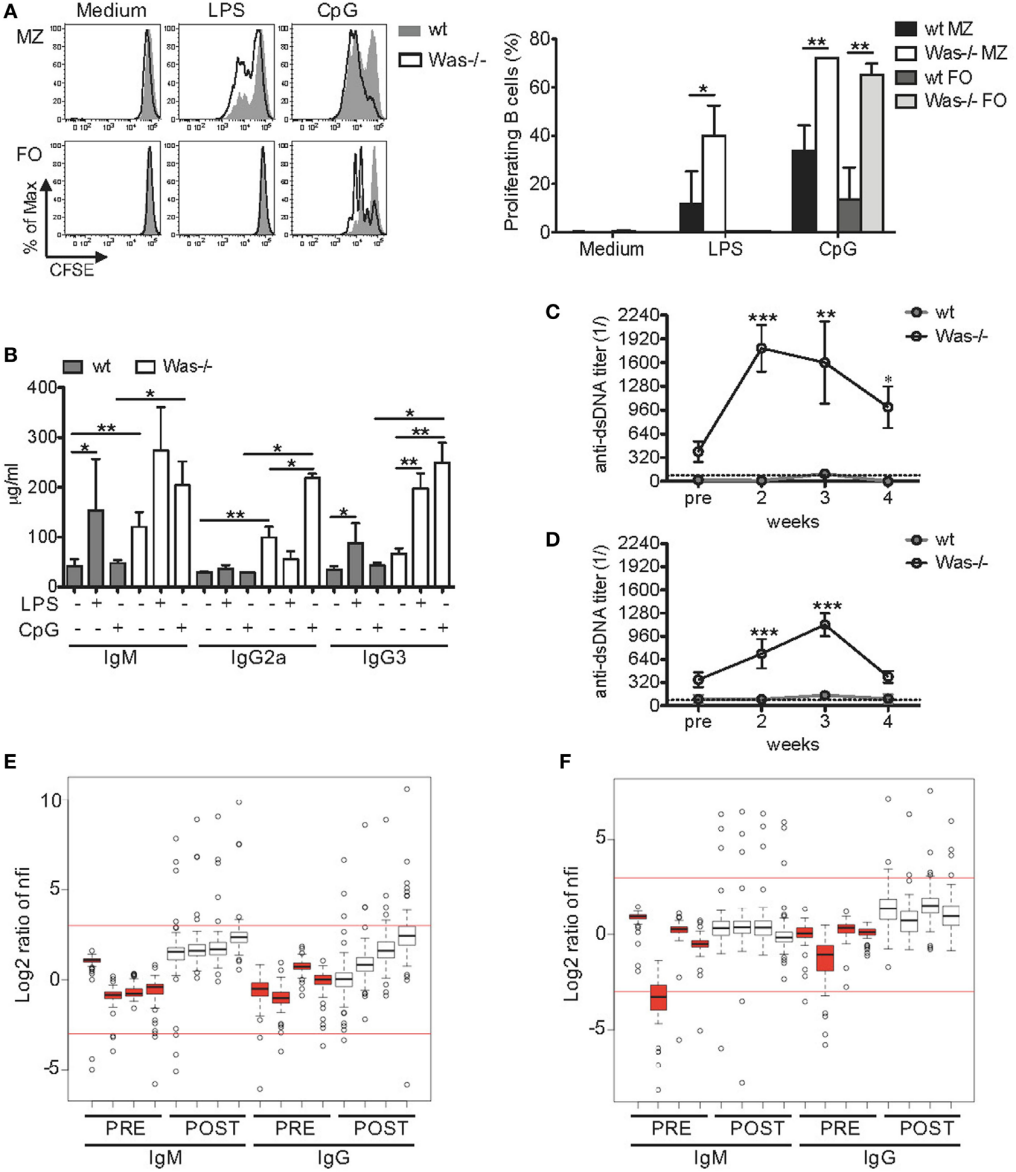
**Increased *in vitro* and *in vivo* response of Wiskott–Aldrich syndrome protein-deficient B cells to Toll-like receptor agonists**. **(A)** Proliferation capacity was evaluated by CFSE dilution assay in sorted marginal zone (MZ) and follicular (FO) B cells isolated from spleen of wild-type (wt) and *Was*^−/−^ mice and stimulated for 72 h with LPS (1 µg/mL), CpG (1 µg/mL), or medium alone. Representative histograms are shown in the left panel, and mean values ±SD are indicated in the bar graphs (three independent experiments). Significant differences are calculated with two-way analysis of variance (ANOVA) (**P < 0.01, and *P < 0.05). **(B)** Total immunoglobulin (Ig) M, IgG2a, and IgG3 levels were measured in the sera of wt and *Was*^−/−^ mice receiving repeated injections of LPS, CpG, or PBS. Statistical differences were analyzed with the Mann–Whitney test (**P < 0.005 and *P < 0.05). **(C,D)** Kinetics of serum titers of anti-double-stranded DNA (dsDNA) antibodies circulating in wt and *Was*^−/−^ mice after *in vivo* administration of LPS **(C)** or CpG **(D)** were evaluated by ELISA. Dotted lines indicate the serum titer considered negative for anti-dsDNA antibodies. Statistical differences were evaluated with two-way ANOVA (***P < 0.001, **P < 0.01, and *P < 0.05). **(E,F)** The positivity of serum IgM or IgG antibodies to 74 autoantigens was analyzed in the sera of *Was*^−/−^ mice (*n* = 4) by an autoantibody array after *in vivo* administration of LPS **(E)** or CpG **(F)**. The signal intensity of the autoantibodies before (PRE, red) and after (POST, white) the treatments was normalized for the background fluorescence, and the normalized fluorescence intensities (nfis) are shown as log2 ratio as respect to the average nfi of PRE *Was*^−/−^ mice.

Given the increased production of total Igs and autoantibodies, we questioned whether the response of *Was*^−/−^ B cells to *in vitro* stimuli could also be altered. We thus evaluated whether the response to TLRs and their ligands, important regulators of B cell functions (32), was dysfunctional in *Was*^−/−^ B cells. To this aim, we tested the proliferative response of mature splenic B cells to LPS (TLR4 ligand) and CpG-containing DNAs (TLR9 ligand). To avoid biases in the proliferation assays, due to the well-known perturbation of the mature B cell subsets in the spleen of *Was*^−/−^ mice with a marked reduction of MZ B cells (11, 27), the experiments were performed on sorted MZ and FO B cells. As expected, both wt and *Was*^−/−^ FO B cells were unresponsive to low doses of LPS stimulation (33, 34), while stimulation with CpG resulted in a higher proliferation of FO B cells from *Was*^−/−^ mice compared to wt mice (Figure 2A). *Was*^−/−^ MZ B cells showed a significantly higher proliferation than wt MZ B cells to both TLR4 and TLR9 stimulations. We did not find differences in the expression of TLR4 and TLR9 in *Was*^−/−^ FO and MZ B cells compared to wt B cells (data not shown). These results indicate that *Was*^−/−^ B cells are hyperproliferating to TLR-dependent stimuli when compared to wt B cells.

Improper activation of TLRs could favor the expansion of autoreactive cells (35). To assess the role played by pattern recognition receptors in WAS autoimmunity, we evaluated the *in vivo* response of *Was*^−/−^ mice to repeated TLR stimulations, a condition mimicking chronic inflammatory state. In fact, in WAS, it has been postulated that a chronic inflammatory state, due to an incomplete pathogen clearance, could disrupt immunological tolerance. We treated wt and *Was*^−/−^ mice by i.v. injections of LPS once a week or by i.p. injections of CpG twice a week for 4 weeks. In parallel, control groups for wt and *Was*^−/−^ mice received PBS. All animals were sacrificed 1 week after the last dose (scheme of treatment is summarized in Figure S1A in Supplementary Material). At the time of sacrifice, both LPS and CpG treatments caused a mild change in the amount of circulating Igs in wt mice, with only an increase in IgM and IgG3 in response to LPS (Figure 2B). On the contrary, LPS and CpG treatments significantly affected the amount of circulating Igs in *Was*^−/−^ mice (Figure 2B). In particular, IgG3 was increased in response to LPS and IgG2a and IgG3 in response to CpG. Moreover, the levels of IgM, IgG2a, and IgG3 were all significantly higher in *Was*^−/−^ mice after CpG treatment, compared to wt mice (Figure 2B). To evaluate the type of Igs circulating in treated mice, we monitored the kinetics of autoantibody production by measuring the levels of antibodies anti-dsDNA every week starting at 2 weeks after the first dose. In wt mice, the titers of anti-dsDNA antibodies were under or just above the threshold of positivity at all time points analyzed (Figures 2C,D). On the contrary, *Was*^−/−^ mice showed high autoantibody titers, starting at 2 weeks from the first dose, which remained high until the end of treatment with both LPS and CpG (Figures 2C,D). Interestingly, LPS administration induced a more rapid production of autoantibodies, reaching titers higher than those achieved upon CpG administration. On the basis of these results, we analyzed the profile of circulating autoantibodies by performing an autoantibody array on the sera of *Was*^−/−^ mice before and after the *in vivo* administrations of LPS and CpG to screen the positivity of IgM or IgG antibodies to 74 autoantigens (30). We noticed that *Was*^−/−^ mice, injected with LPS or CpG, showed high normalized fluorescence intensity (nfi) values for many autoantigens tested with several outliers above the cutoff of +3 (which corresponds to a fold increase of 8 compared to the nfi before treatment) (Figures 2E,F). The specificity and the signal intensity of autoantibodies detected in both IgM and IgG subclasses are represented in the heat maps of LPS- and CpG-treated groups (Figures S2A,B in Supplementary Material, respectively). LPS injections in *Was*^−/−^ mice led to an increased production of autoantibodies belonging to both IgM and IgG isotypes, even though the latter did not reach statistical significance (*P* = 0.0571) (Figure S2A in Supplementary Material). Instead, both IgM and, even more, IgG autoantibodies were significantly increased after *in vivo* CpG administration in *Was*^−/−^ mice (Figure S2B in Supplementary Material). The specificity of autoantibodies produced by *Was*^−/−^ mice challenged with LPS or CpG covered a broad panel of autoantigens that included antinuclear antibodies (with anti-dsDNA, anti-histone H2a or H3, anti-KU, antiribonucleoprotein complex, and antitopoisomerase Scl70), antineutrophil cytoplasmic antibodies [anti-bactericidal permeability-increasing protein (BPI), antiglomerular basement membrane, antilaminin, antimatrigel], anticollagen antibodies, and organ-specific autoantibodies such as antithyroglobulin protein (found in thyroid cells) or the liver-specific antiliver cytosol. In particular, three autoantibodies, of both IgM and IgG subclasses, were produced in *Was*^−/−^ mice independently of the treatment: BPI (antimicrobial protein that binds and neutralizes LPS of Gram-negative bacteria) (36), anti-Ku antibody (a rare type of antinuclear antibody typical of myositis) (37), and anti-SRP54 (signal recognition particle involved in targeting secretory proteins to the rough endoplasmic reticulum membrane) (38).

**Figure 3:**
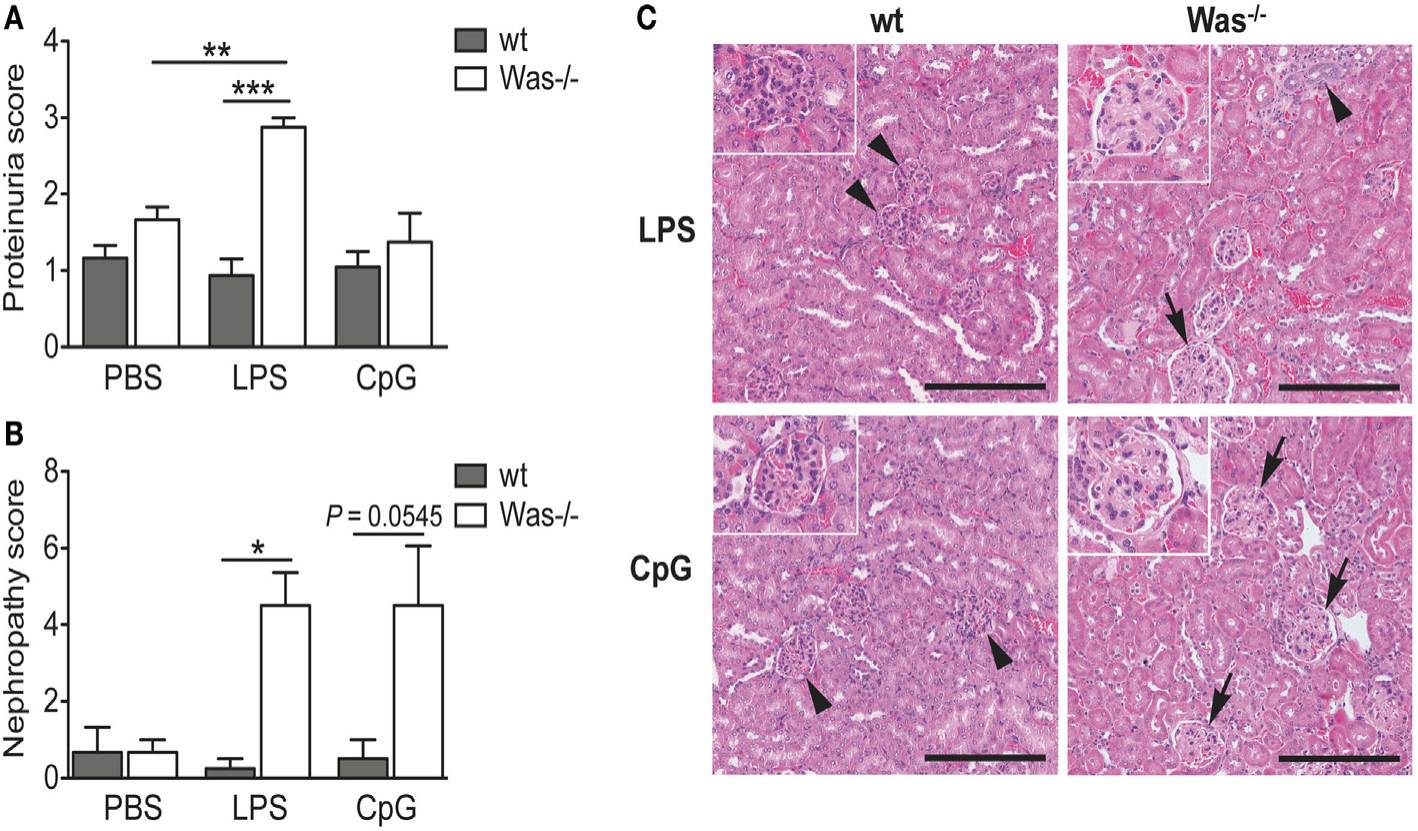
**Toll-like receptor-induced kidney damage in *Was^−/−^* mice**. **(A)** Proteinuria was determined at the time of sacrifice of mice treated with PBS, LPS, or CpG (*n* = 5 per group), and the score was assigned starting from 0 (absence or trace of proteins, <30 mg/dL) to 4 (heavy proteinuria, >2,000 mg/dL). **(B)** The nephropathy score was based on the frequency and the severity of different renal lesions observed in wild-type (wt) and *Was*^−/−^ mice (*n* = 5 per group) and ranged from 0, with no lesions, to 4, with severe histopathological changes. Mean values and SEM are reported in the graphs. The Mann–Whitney test was applied to assess whether differences between wt and *Was*^−/−^ were significant (***P < 0.0001, **P < 0.005, and *P < 0.05). **(C)** Hematoxylin and eosin staining of kidney sections of wt and *Was*^−/−^ mice after LPS or CpG treatment. Unaffected renal parenchyma with normal corpuscles is shown in wt mice (arrowheads). Glomerulopathy (arrows) characterized by segmental to global hyaline thickening of glomerular basement membrane with hypertrophic cells populating the affected mesangiocapillary areas was present in *Was*^−/−^ mice. Scale bar = 200 µm (main panel) and 100 µm (inset).

To evaluate the presence of tissue damage caused by the chronic treatment with LPS and CpG, we analyzed the proteinuria of mice and found an increased proteinuria index in *Was*^−/−^ mice receiving LPS compared to wt mice (Figure 3A). This difference was not present after CpG administration (Figure 3A). To validate these data, we performed a histopathological examination of kidney sections. Microscopic lesions observed were classified according to the INHAND system, and a nephropathy score was assigned as described in Section “Material and Methods.” As shown in Figure 3B, LPS administration caused a significant increase in frequency and/or severity of renal lesions as compared to wt mice. Also the *Was*^−/−^ group treated with CpG showed a high nephropathy score, although it was not statistically different from wt (*P* = 0.0545). We could observe a glomerulopathy in *Was*^−/−^ mice challenged with both LPS and CpG, while the renal parenchyma was unaffected and presented normal renal corpuscles in treated wt mice (Figure 3C).

Altogether, these data demonstrate that *in vivo* TLR4 and TLR9 stimulations trigger activation of autoreactive B cells leading to increased production of autoantibodies and renal damage in *Was*^−/−^ mice.

### *In Vivo* Response to Challenge with Apoptotic Cells

**Figure 4:**
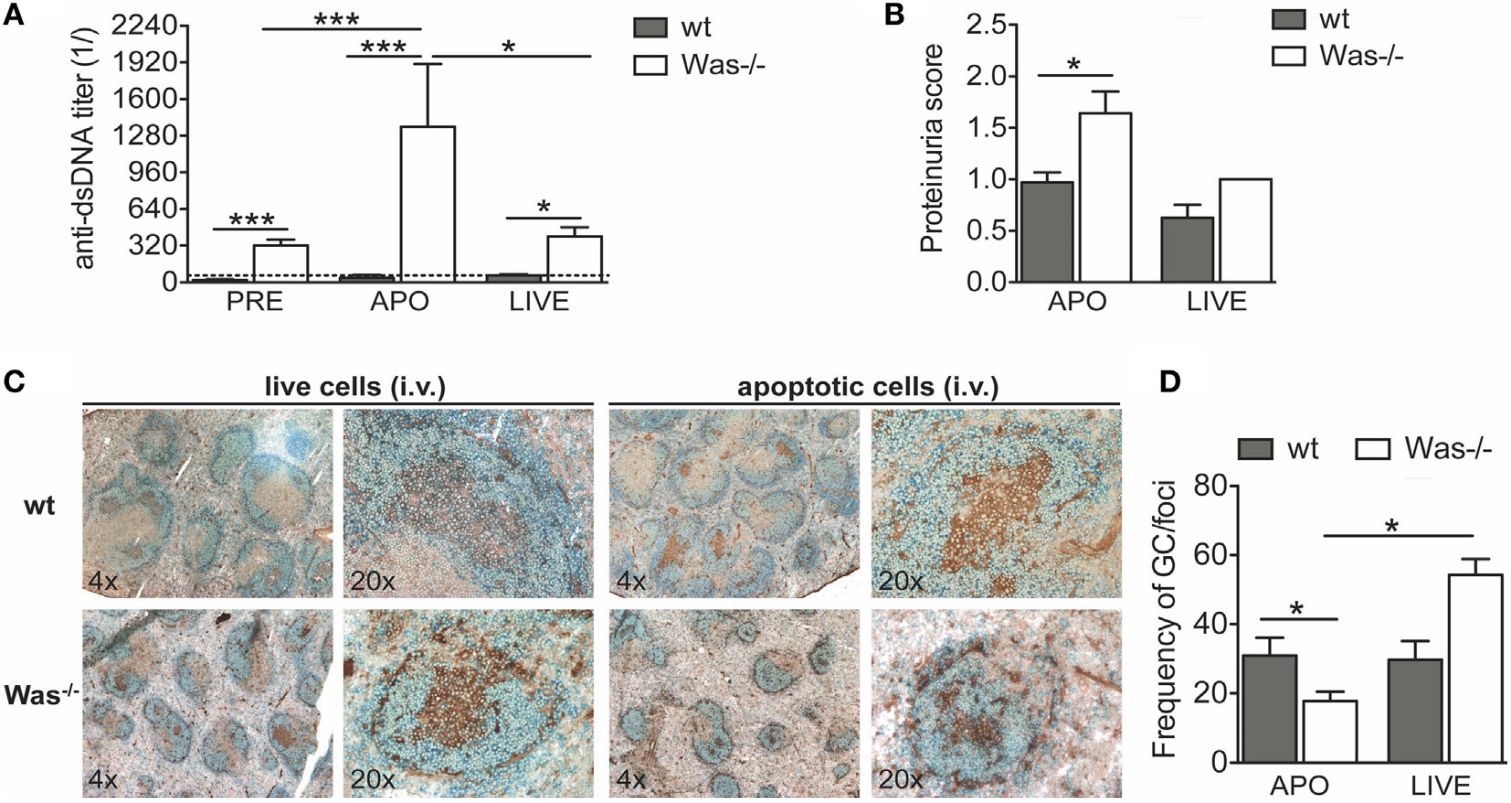
***In vivo* challenge with apoptotic cells triggered autoreactive B cells and kidney damage in *Was^−/−^* mice**. **(A)** Serum titers of anti-double-stranded DNA (dsDNA) circulating antibodies in wild-type (wt) and *Was*^−/−^ mice evaluated by ELISA before (PRE, *n* = 16 per group) and 8 weeks after the first injection with apoptotic (APO, *n* = 7–8 per group) or live cells (LIVE, *n* = 4 per group). **(B)** Proteinuria scores were determined at the time of the sacrifice. **(C)** Splenic sections of wt and *Was*^−/−^ mice treated with live or apoptotic cells were stained with peanut agglutinin (PNA) (brown) and B220 Ab (blue). **(D)** The frequency of germinal centers on the total number of foci was evaluated by immunohistochemistry analysis of splenic sections stained with PNA and B220 Ab. Mean values ±SD are reported in the graphs. Mann–Whitney test was applied to assess the significant differences between wt and *Was*^−/−^ groups (***P < 0.0001 and *P < 0.05).

An antigen overload in immunodeficient conditions could trigger development of autoimmunity. To test the effect of an overload of apoptotic cells on the development of autoimmunity in *Was*^−/−^ mice, we i.v. injected syngeneic apoptotic cells in wt and *Was*^−/−^ mice, once a week for 4 weeks, to induce an overload of self-antigens. Live cells were injected in control groups. Seven weeks after the last administration of apoptotic cells, we sacrificed the mice and examined the response to apoptotic cell injections in terms of levels of autoantibodies against dsDNA and proteinuria (schematic representation of experimental protocol is shown in Figure S1B in Supplementary Material). We observed that *Was*^−/−^ mice injected with apoptotic cells produced significantly higher levels of anti-dsDNA antibodies compared with wt mice treated with apoptotic cells or with the same group before treatment (Figure 4A). The reaction was specific to apoptotic cells, since in mice injected with live cells, the autoantibody levels were higher than in wt mice, but similar to those of *Was*^−/−^ mice before treatment. In addition, the increased response to apoptotic cells was associated with an increased proteinuria, suggestive of kidney tissue damage, only in *Was*^−/−^ mice injected with apoptotic cells (Figure 4B). Immunohistochemical analysis of splenic sections stained with B220 and peanut agglutinin showed a reduced frequency of germinal centers on the total number of foci in *Was*^−/−^ mice injected with apoptotic cells compared to wt mice challenged with apoptotic cells or *Was*^−/−^ mice injected with live cells, likely indicating different kinetics of germinal center formation between wt and *Was*^−/−^ mice (Figures 4C,D).

These results demonstrate that *Was*^−/−^ B cells have a lower activation threshold than wt B cells and that an overload of self-antigens is sufficient to overcome tolerance mechanisms.

### *In Vivo* Response to Viral Infection

**Figure 5:**
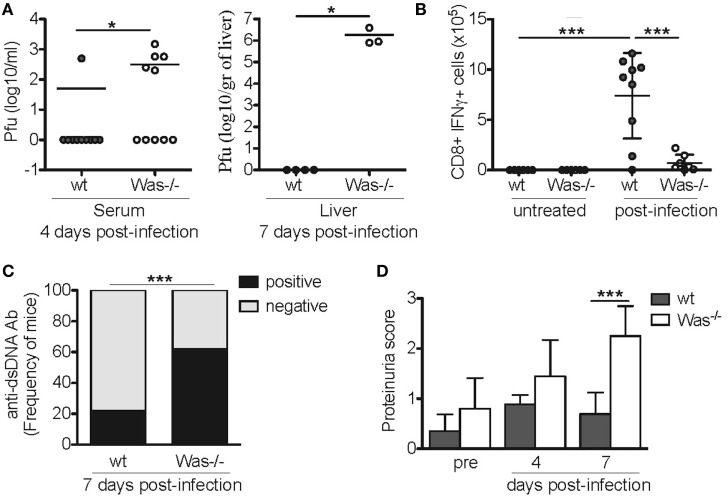
**Lymphocytic choriomeningitis virus (LCMV) infection in *Was^−/−^* mice**. **(A)** Quantification by plaque assay of the viral titers in the serum (left panel) and liver (right panel) of wild-type (wt) and *Was*^−/−^ mice at the indicated time points after LCMV arm infection (10^6^ p.f.u./mouse). Mann–Whitney test was applied to assess the significant differences between wt and *Was*^−/−^ groups (*P < 0.05). **(B)** Assessment by intracellular IFNγ staining of the absolute number of GP33-specific CD8^+^ T cells recovered from the spleens of infected animals at the time of sacrifice. Mean values ±SD are reported in the graphs, and significant differences between wt and *Was*^−/−^ groups were evaluated by one-way ANOVA test (***P < 0.0001). **(C)** Graph shows the percentage of mice positive or negative for circulating autoantibodies against double-stranded DNA (dsDNA) after LCMV arm infection, evaluated in the sera by ELISA (*n* = 10 mice per group). Significant differences were statistically calculated by χ^2^ test (***P < 0.0005). **(D)** Proteinuria scores were assessed before (pre) and 4 and 7 days after LCMV arm infection. Two-way ANOVA test was applied to assess the significant differences between wt and *Was*^−/−^ groups (***P < 0.001, *n* = 10 mice per group).

To test whether also incomplete pathogen clearance following viral infection could disrupt immunological tolerance and trigger development of autoimmunity, we performed acute LCMV infection in *Was*^−/−^ mice. wt and *Was*^−/−^ mice were infected i.v. with 1 × 10^6^ p.f.u. LCMV (Armstrong strain). Mice were monitored for 7 days and then sacrificed. We observed a 30% mortality in *Was*^−/−^ mice (data not shown), as they were unable to completely clear the infection. In fact, higher viral titers were detected in the serum of mutant mice at day 4 in the serum and at day 7 in the liver (Figure 5A). Consistently, *in vitro* stimulation of CD8^+^ T cells obtained from the spleens of infected mice with GP33-specific LCMV peptide revealed a decreased CD8-mediated specific response to the virus, as shown by the reduced production of IFNγ in *Was*^−/−^ mice (Figure 5B). We then evaluated the effect of acute LCMV infection on the generation of autoantibodies. Serum from LCMV-infected *Was*^−/−^ mice showed a significantly increased positivity for anti-dsDNA antibodies compared to wt animals (Figure 5C). Finally, we evaluated renal tissue damage by dosing proteinuria, and we detected a significant increase of proteinuria score at day 7 after infection, only in *Was*^−/−^ mice (Figure 5D).

Taken together, our results indicate that incomplete clearance of viral infections can lead to a break of tolerance and trigger an autoimmune-like response in *Was*^−/−^ mice.

### Increased Production of Pro-inflammatory Cytokines in *Was*^−/−^ Mice after *In Vivo* Chronic Stimulations

**Figure 6:**
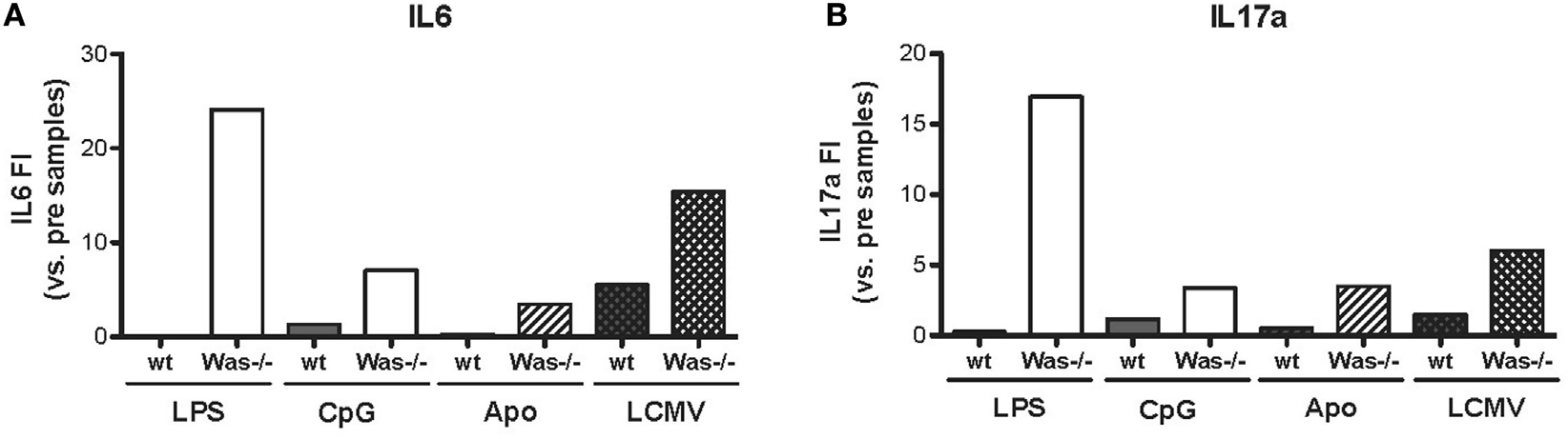
**Cytokine production in *Was^−/−^* mice after *in vivo* chronic stimulations**. The fold increase of IL6 **(A)** and IL17a **(B)** concentrations was calculated as the ratio between the mean value of cytokine production after the indicated *in vivo* treatment over the mean value before the treatment for each group of mice [LPS/CpG, *n* = 4 mice; Apo, *n* = 7–8 mice; lymphocytic choriomeningitis virus (LCMV), *n* = 5 mice].

We performed *in vivo* chronic stimulation in *Was*^−/−^ mice by means of administration of TLR agonists (LPS and CpG), injection of apoptotic cells, and LCMV infection. In all cases, we observed the increase of autoantibody production and the development of signs of autoimmunity, which could indicate a common mechanism of peripheral break of tolerance induction in *Was*^−/−^ mice. To this end, we tested whether chronic stimulations might induce an inflammatory state that could trigger the activation of potentially autoreactive B cells in *Was*^−/−^ mice by evaluating the production of pro-inflammatory cytokines (IL6, IL17a, IFNg, IL1b, IL10, and TNFa) in the serum of animals, before and after *in vivo* chronic stimulations. As shown in Figure 6, production of both IL6 and IL17a was strongly increased in *Was*^−/−^ mice, compared to the wt counterpart, after all different chronic stimulations (LPS, CpG, apoptotic cells, and LCMV infection). Upon LPS administration, also IFNg production was increased in *Was*^−/−^ mice (Figure S3 in Supplementary Material). No difference in the production of the other cytokines evaluated (IL1b, IL10, and TNFa) was found between wt and *Was*^−/−^ mice (data not shown).

## Discussion

Many cellular defects contributing to the immunopathology of WAS have been identified over the years; however, the mechanisms leading to the development of autoimmunity still remain not completely understood. In particular, although several groups have demonstrated that the B cell intrinsic defect contributes to WAS-related autoimmunity (12, 18, 19, 39, 40), the pathogenic mechanisms are still matter of study. In this article, we took advantage of the *Was*^−/−^ mouse model to gain new insights in the mechanisms responsible for the humoral autoimmunity observed in the absence of WASp.

In naïve *Was*^−/−^ mice, we found high levels of circulating Igs and increased frequency of Ig-secreting cells, plasma cells, and GC B cells (data not shown), suggesting a critical role for WASp in the differentiation toward Ig-secreting cells, both *in vitro* and *ex vivo*, in line with results previously published (19, 41, 42). *Was*^−/−^ mice are also characterized by the presence of autoreactive B cells, which produce autoantibodies against dsDNA, tissue antigens, and platelets. Antinuclear and anti-dsDNA antibodies were initially described in WAS by Humblet-Baron et al. (6) and later observed by other groups in *Was*^−/−^ mice with different gene targeting strategies and/or genetic mutations/backgrounds (17–19, 39). Thus, the presence of autoantibodies is a consistent finding of *Was*^−/−^ mice, often without any overt autoimmune manifestations. In fact, only the 129Sv *Was*^−/−^ strain develops spontaneous, radiation-induced colitis, which resembles autoimmune inflammatory bowel disease (43), and IgA nephropathy-like glomerulonephritis (44, 45).

We showed a hyperactivation of WASp-negative B cells to TLR-dependent stimuli both *in vitro* and *in vivo*. Alterations in the B cell cytoskeleton of *Was*^−/−^ mice could influence their activation threshold, and, in this respect, it is known that membrane lipid rafts participate in many of the cell surface events involved in B cell activation, including signaling by the BCR, endocytosis of antigen bound to the BCR, and TLR activation (46, 47). We know that WASp is required for the dynamics of lipid rafts, which are necessary for immunological synapse assembly and amplification of downstream signals during T-cell activation (48, 49). Thus, it is likely that an alteration of the movements of lipid rafts in WASp-negative B cells could sustain the abnormal responses to TLR engagement.

It has been demonstrated that MyD88 signaling is essential for development of systemic autoimmune disease in *Was*^−/−^ chimeras (18). Accordingly, we observed that *in vivo* triggering of TLRs stimulates the production of anti-dsDNA autoantibodies with renal damage in *Was*^−/−^ mice, demonstrating for the first time the *in vivo* hyperresponsiveness of WASp-negative B cells to TLR ligands. These findings suggest that, in the absence of WASp, improper activation of TLRs could favor expansion of autoreactive B cells, as also observed in SLE (50). DNA ligands for TLR9 may be provided *in vivo* by apoptotic bodies that are incompletely cleared and could lead to uncontrolled activation of the TLR9-MyD88 pathway promoting anti-DNA autoantibody generation (51, 52). Delayed phagocytosis of apoptotic cells has been reported in WASp deficiency (23) and proposed as another potential mechanism of loss of peripheral tolerance (53). We indeed demonstrated here that an overload of apoptotic cells perturbs the tolerance threshold resulting in high autoantibodies titers in *Was*^−/−^ mice. In particular, the overload of apoptotic cells activates autoreactive B cells, which have increased access to self-antigens derived from apoptotic cells, thus inducing the increased production of autoantibodies in *Was*^−/−^ mice. However, we detected smaller GC in *Was*^−/−^ mice after injection of apoptotic cells, compared to wt mice, differently from what has been described in literature (41). We believe that there is no discrepancy with our results, since we evaluated GC at a later time point compared to the article by Dahlberg and colleagues. We think that the germinal centers in our mice were past their maximum expansion, and the fact that in *Was*^−/−^ mice they were even smaller than in wt mice could indicate that they were more exhausted due to the extensive Ig production and proliferation typical of *Was*^−/−^ mice after *in vivo* stimuli.

Along the same line, we tested whether the infection of *Was*^−/−^ mice with viral pathogens, e.g., LCMV, could trigger the development of autoimmunity. Indeed, *Was*^−/−^ mice infected with LCMV showed a defective clearance of the virus, and the resulting chronic exposure to viral antigens or the persistence of an inflammatory state led to the development of signs of autoimmunity, with an increased production of anti-dsDNA autoantibodies and the presence of renal tissue damage.

In summary, all chronic stimulations tested in *Was*^−/−^ mice were able to generate similar phenotypes, characterized by an increased production of autoantibodies and signs of autoimmunity, including proteinuria and renal tissue damage. Thus, we hypothesize that a similar mechanism of break of tolerance might take place in *Was*^−/−^ mice. Indeed, we saw that all treatments led to an overproduction of pro-inflammatory cytokines, such as IL6 and IL17a, only in *Was*^−/−^ mice and not in wt animals. These results indicate that the chronic stimulations were inducing a persistent inflammatory state able to promote the expansion of the potentially autoreactive B cells present in *Was*^−/−^ mice, allowing them escape tolerance mechanisms and become pathogenic. These mechanisms, together with defective T cell tolerance present in WAS, could add an additional layer of immune dysregulation contributing to development of autoimmunity in WAS.

## Ethics Statement

This study was carried out in accordance with the recommendations of the Italian Ministry of Health. The protocol was approved by the Animal Care and Use Committee of the San Raffaele Scientific Institute (IACUC 406 and 557).

## Author Contributions

MC, MI, ET, AV, and MB designed the experiments. MC, FP, ED, LS, DI, AS, FS, EF, KC-L, and ER performed the experiments. MC, PU, FB, PP, MI, ET, AV, and MB analyzed the data. MI and ET revised the manuscript. MC, AV, and MB wrote the manuscript.

## Conflict of Interest Statement

The authors declare that the research was conducted in the absence of any commercial or financial relationships that could be construed as a potential conflict of interest.

## References

[1] Ochs HD Thrasher AJ. The Wiskott-Aldrich syndrome. J Allergy Clin Immunol (2006) 117(4):725–38. 10.1016/j.jaci.2006.02.005 16630926

[2] Stewart DM Treiber-Held S Kurman CC Facchetti F Notarangelo LD Nelson DL. Studies of the expression of the Wiskott-Aldrich syndrome protein. J Clin Invest (1996) 97(11):2627–34. 10.1172/JCI118712 8647957 PMC507350

[3] Symons M Derry JM Karlak B Jiang S Lemahieu V McCormick F Wiskott-Aldrich syndrome protein, a novel effector for the GTPase CDC42Hs, is implicated in actin polymerization. Cell (1996) 84(5):723–34. 10.1016/S0092-8674(00)81050-8 8625410

[4] Ozsahin H Cavazzana-Calvo M Notarangelo LD Schulz A Thrasher AJ Mazzolari E Long-term outcome following hematopoietic stem-cell transplantation in Wiskott-Aldrich syndrome: collaborative study of the European Society for Immunodeficiencies and European Group for Blood and Marrow Transplantation. Blood (2008) 111(1):439–45. 10.1182/blood-2007-03-076679 17901250

[5] Adriani M Aoki J Horai R Thornton AM Konno A Kirby M Impaired in vitro regulatory T cell function associated with Wiskott-Aldrich syndrome. Clin Immunol (2007) 124(1):41–8. 10.1016/j.clim.2007.02.001 17512803 PMC1986664

[6] Humblet-Baron S Sather B Anover S Becker-Herman S Kasprowicz DJ Khim S Wiskott-Aldrich syndrome protein is required for regulatory T cell homeostasis. J Clin Invest (2007) 117(2):407–18. 10.1172/JCI29539 17218989 PMC1764857

[7] Maillard MH Cotta-de-Almeida V Takeshima F Nguyen DD Michetti P Nagler C The Wiskott-Aldrich syndrome protein is required for the function of CD4(+)CD25(+)Foxp3(+) regulatory T cells. J Exp Med (2007) 204(2):381–91. 10.1084/jem.20061338 17296786 PMC2118715

[8] Marangoni F Trifari S Scaramuzza S Panaroni C Martino S Notarangelo LD WASP regulates suppressor activity of human and murine CD4(+)CD25(+)FOXP3(+) natural regulatory T cells. J Exp Med (2007) 204(2):369–80. 10.1084/jem.20061334 17296785 PMC2118740

[9] Adriani M Jones KA Uchiyama T Kirby MR Silvin C Anderson SM Defective inhibition of B-cell proliferation by Wiskott-Aldrich syndrome protein-deficient regulatory T cells. Blood (2011) 117(24):6608–11. 10.1182/blood-2010-12-322834 21515824 PMC3123025

[10] Westerberg L Greicius G Snapper SB Aspenstrom P Severinson E. Cdc42, Rac1, and the Wiskott-Aldrich syndrome protein are involved in the cytoskeletal regulation of B lymphocytes. Blood (2001) 98(4):1086–94. 10.1182/blood.V98.4.1086 11493455

[11] Westerberg L Larsson M Hardy SJ Fernandez C Thrasher AJ Severinson E. Wiskott-Aldrich syndrome protein deficiency leads to reduced B-cell adhesion, migration, and homing, and a delayed humoral immune response. Blood (2005) 105(3):1144–52. 10.1182/blood-2004-03-1003 15383456

[12] Castiello MC Bosticardo M Pala F Catucci M Chamberlain N van Zelm MC Wiskott-Aldrich syndrome protein deficiency perturbs the homeostasis of B-cell compartment in humans. J Autoimmun (2014) 50:42–50. 10.1016/j.jaut.2013.10.006 24369837 PMC4012141

[13] Simon KL Anderson SM Garabedian EK Moratto D Sokolic RA Candotti F Molecular and phenotypic abnormalities of B lymphocytes in patients with Wiskott-Aldrich syndrome. J Allergy Clin Immunol (2014) 133(3):896–9.e4. 10.1016/j.jaci.2013.08.050 24210885 PMC3943658

[14] Castiello MC Scaramuzza S Pala F Ferrua F Uva P Brigida I B-cell reconstitution after lentiviral vector-mediated gene therapy in patients with Wiskott-Aldrich syndrome. J Allergy Clin Immunol (2015) 136(3):692–702.e2. 10.1016/j.jaci.2015.01.035 25792466 PMC4559137

[15] Dupuis-Girod S Medioni J Haddad E Quartier P Cavazzana-Calvo M Le Deist F Autoimmunity in Wiskott-Aldrich syndrome: risk factors, clinical features, and outcome in a single-center cohort of 55 patients. Pediatrics (2003) 111(5 Pt 1):e622–7. 10.1542/peds.111.5.e622 12728121

[16] Schurman SH Candotti F. Autoimmunity in Wiskott-Aldrich syndrome. Curr Opin Rheumatol (2003) 15(4):446–53. 10.1097/00002281-200307000-00012 12819473

[17] Nikolov NP Shimizu M Cleland S Bailey D Aoki J Strom T Systemic autoimmunity and defective Fas ligand secretion in the absence of the Wiskott-Aldrich syndrome protein. Blood (2010) 116(5):740–7. 10.1182/blood-2009-08-237560 20457871 PMC2918330

[18] Becker-Herman S Meyer-Bahlburg A Schwartz MA Jackson SW Hudkins KL Liu C WASp-deficient B cells play a critical, cell-intrinsic role in triggering autoimmunity. J Exp Med (2011) 208(10):2033–42. 10.1084/jem.20110200 21875954 PMC3182055

[19] Recher M Burns SO de la Fuente MA Volpi S Dahlberg C Walter JE B cell-intrinsic deficiency of the Wiskott-Aldrich syndrome protein causes severe abnormalities of the peripheral B-cell compartment in mice. Blood (2012) 119:2819–28. 10.1182/blood-2011-09-379412 22302739 PMC3327460

[20] Marshak-Rothstein A. Toll-like receptors in systemic autoimmune disease. Nat Rev Immunol (2006) 6(11):823–35. 10.1038/nri1957 17063184 PMC7097510

[21] Sullivan KE Mullen CA Blaese RM Winkelstein JA. A multiinstitutional survey of the Wiskott-Aldrich syndrome. J Pediatr (1994) 125(6 Pt 1):876–85. 10.1016/S0022-3476(05)82002-5 7996359

[22] Imai K Morio T Zhu Y Jin Y Itoh S Kajiwara M Clinical course of patients with WASP gene mutations. Blood (2004) 103(2):456–64. 10.1182/blood-2003-05-1480 12969986

[23] Leverrier Y Lorenzi R Blundell MP Brickell P Kinnon C Ridley AJ Cutting edge: the Wiskott-Aldrich syndrome protein is required for efficient phagocytosis of apoptotic cells. J Immunol (2001) 166(8):4831–4. 10.4049/jimmunol.166.8.4831 11290758

[24] Medzhitov R. Recognition of microorganisms and activation of the immune response. Nature (2007) 449(7164):819–26. 10.1038/nature06246 17943118

[25] Zhang J Shehabeldin A da Cruz LA Butler J Somani AK McGavin M Antigen receptor-induced activation and cytoskeletal rearrangement are impaired in Wiskott-Aldrich syndrome protein-deficient lymphocytes. J Exp Med (1999) 190(9):1329–42. 10.1084/jem.190.9.1329 10544204 PMC2195687

[26] Guidotti LG Inverso D Sironi L Di Lucia P Fioravanti J Ganzer L Immunosurveillance of the liver by intravascular effector CD8(+) T cells. Cell (2015) 161(3):486–500. 10.1016/j.cell.2015.03.005 25892224 PMC11630812

[27] Bosticardo M Draghici E Schena F Sauer AV Fontana E Castiello MC Lentiviral-mediated gene therapy leads to improvement of B-cell functionality in a murine model of Wiskott-Aldrich syndrome. J Allergy Clin Immunol (2011) 127(6):1376–84.e5. 10.1016/j.jaci.2011.03.030 21531013

[28] Sauer AV Brigida I Carriglio N Hernandez RJ Scaramuzza S Clavenna D Alterations in the adenosine metabolism and CD39/CD73 adenosinergic machinery cause loss of Treg cell function and autoimmunity in ADA-deficient SCID. Blood (2012) 119(6):1428–39. 10.1182/blood-2011-07-366781 22184407 PMC3426348

[29] Radaelli E Del Piero F Aresu L Sciarrone F Vicari N Mattiello S Expression of major histocompatibility complex class II antigens in porcine leptospiral nephritis. Vet Pathol (2009) 46(5):800–9. 10.1354/vp.08-VP-0078-R-FL 19179617

[30] Li QZ Zhou J Wandstrat AE Carr-Johnson F Branch V Karp DR Protein array autoantibody profiles for insights into systemic lupus erythematosus and incomplete lupus syndromes. Clin Exp Immunol (2007) 147(1):60–70. 10.1111/j.1365-2249.2006.03251.x 17177964 PMC1810453

[31] Goeman JJ van de Geer SA van Houwelingen HC Testing against a high dimensional alternative. J R Stat Soc Series B Stat Methodol (2006) 68:477–93. 10.1111/J.1467-9868.2006.00551.X

[32] Peng SL. Signaling in B cells via toll-like receptors. Curr Opin Immunol (2005) 17(3):230–6. 10.1016/j.coi.2005.03.003 15886111

[33] Snapper CM Yamada H Smoot D Sneed R Lees A Mond JJ. Comparative in vitro analysis of proliferation, Ig secretion, and Ig class switching by murine marginal zone and follicular B cells. J Immunol (1993) 150(7):2737–45. 7681079

[34] Oliver AM Martin F Gartland GL Carter RH Kearney JF. Marginal zone B cells exhibit unique activation, proliferative and immunoglobulin secretory responses. Eur J Immunol (1997) 27(9):2366–74. 10.1002/eji.1830270935 9341782

[35] Green NM Marshak-Rothstein A Toll-like receptor driven B cell activation in the induction of systemic autoimmunity. Semin Immunol (2011) 23(2):106–12. 10.1016/j.smim.2011.01.016 21306913 PMC3070769

[36] Schinke S Fellermann K Herlyn K Reichel PH Fundke R Stange EF Autoantibodies against the bactericidal/permeability-increasing protein from inflammatory bowel disease patients can impair the antibiotic activity of bactericidal/permeability-increasing protein. Inflamm Bowel Dis (2004) 10(6):763–70. 10.1097/00054725-200411000-00011 15626895

[37] Belizna C Henrion D Beucher A Lavigne C Ghaali A Levesque H. Anti-Ku antibodies: clinical, genetic and diagnostic insights. Autoimmun Rev (2010) 9(10):691–4. 10.1016/j.autrev.2010.05.020 20621654

[38] Romisch K Miller FW Dobberstein B High S. Human autoantibodies against the 54 kDa protein of the signal recognition particle block function at multiple stages. Arthritis Res Ther (2006) 8(2):R39. 10.1186/ar1895 16469117 PMC1526608

[39] Kolhatkar NS Brahmandam A Thouvenel CD Becker-Herman S Jacobs HM Schwartz MA Altered BCR and TLR signals promote enhanced positive selection of autoreactive transitional B cells in Wiskott-Aldrich syndrome. J Exp Med (2015) 212(10):1663–77. 10.1084/jem.20150585 26371186 PMC4577851

[40] Pala F Morbach H Castiello MC Schickel JN Scaramuzza S Chamberlain N Lentiviral-mediated gene therapy restores B cell tolerance in Wiskott-Aldrich syndrome patients. J Clin Invest (2015) 125(10):3941–51. 10.1172/JCI82249 26368308 PMC4607131

[41] Dahlberg CI Torres ML Petersen SH Baptista MA Keszei M Volpi S Deletion of WASp and N-WASp in B cells cripples the germinal center response and results in production of IgM autoantibodies. J Autoimmun (2015) 62:81–92. 10.1016/j.jaut.2015.06.003 26143192 PMC6245946

[42] Volpi S Santori E Abernethy K Mizui M Dahlberg CI Recher M N-WASP is required for B-cell-mediated autoimmunity in Wiskott-Aldrich syndrome. Blood (2016) 127(2):216–20. 10.1182/blood-2015-05-643817 26468226 PMC4713162

[43] Snapper SB Rosen FS Mizoguchi E Cohen P Khan W Liu CH Wiskott-Aldrich syndrome protein-deficient mice reveal a role for WASP in T but not B cell activation. Immunity (1998) 9(1):81–91. 10.1016/S1074-7613(00)80590-7 9697838

[44] Shimizu M Nikolov NP Ueno K Ohta K Siegel RM Yachie A Development of IgA nephropathy-like glomerulonephritis associated with Wiskott-Aldrich syndrome protein deficiency. Clin Immunol (2012) 142(2):160–6. 10.1016/j.clim.2011.10.001 22079330 PMC3273668

[45] Nguyen DD Muthupalani S Goettel JA Eston MA Mobley M Taylor NS Colitis and colon cancer in WASP-deficient mice require helicobacter species. Inflamm Bowel Dis (2013) 19(10):2041–50. 10.1097/Mib.0b013e318295fd8f 23820270 PMC4082694

[46] Triantafilou M Miyake K Golenbock DT Triantafilou K. Mediators of innate immune recognition of bacteria concentrate in lipid rafts and facilitate lipopolysaccharide-induced cell activation. J Cell Sci (2002) 115:2603–11. 12045230 10.1242/jcs.115.12.2603

[47] Gupta N DeFranco AL. Lipid rafts and B cell signaling. Semin Cell Dev Biol (2007) 18(5):616–26. 10.1016/j.semcdb.2007.07.009 17719248 PMC2169358

[48] Dupre L Aiuti A Trifari S Martino S Saracco P Bordignon C Wiskott-Aldrich syndrome protein regulates lipid raft dynamics during immunological synapse formation. Immunity (2002) 17(2):157–66. 10.1016/S1074-7613(02)00360-6 12196287

[49] Kumari S Depoil D Martinelli R Judokusumo E Carmona G Gertler FB Actin foci facilitate activation of the phospholipase C-gamma in primary T lymphocytes via the WASP pathway. Elife (2015) 4:e04953 10.7554/eLife.04953 PMC4355629 25758716

[50] Ehlers M Fukuyama H McGaha TL Aderem A Ravetch JV. TLR9/MyD88 signaling is required for class switching to pathogenic IgG2a and 2b autoantibodies in SLE. J Exp Med (2006) 203(3):553–61. 10.1084/jem.20052438 16492804 PMC2118244

[51] Leadbetter EA Rifkin IR Hohlbaum AM Beaudette BC Shlomchik MJ Marshak-Rothstein A. Chromatin-IgG complexes activate B cells by dual engagement of IgM and toll-like receptors. Nature (2002) 416(6881):603–7. 10.1038/416603a 11948342

[52] Christensen SR Kashgarian M Alexopoulou L Flavell RA Akira S Shlomchik MJ. Toll-like receptor 9 controls anti-DNA autoantibody production in murine lupus. J Exp Med (2005) 202(2):321–31. 10.1084/jem.20050338 16027240 PMC2212997

[53] Westerberg LS Klein C Snapper SB Breakdown of T cell tolerance and autoimmunity in primary immunodeficiency – lessons learned from monogenic disorders in mice and men. Curr Opin Immunol (2008) 20(6):646–54. 10.1016/j.coi.2008.10.004 18955138 PMC2605935

