# Supplementary material for: In Vivo Chronic Stimulation Unveils Autoreactive Potential of Wiskott–Aldrich Syndrome Protein-Deficient B Cells
Source: Front Immunol. 2017 May 2;8:490. doi: 10.3389/fimmu.2017.00490 (PMC5411424; doi:10.3389/fimmu.2017.00490)
Supplement: Supplementary file 1 [file Presentation_1.PPTX]

## Slide 1
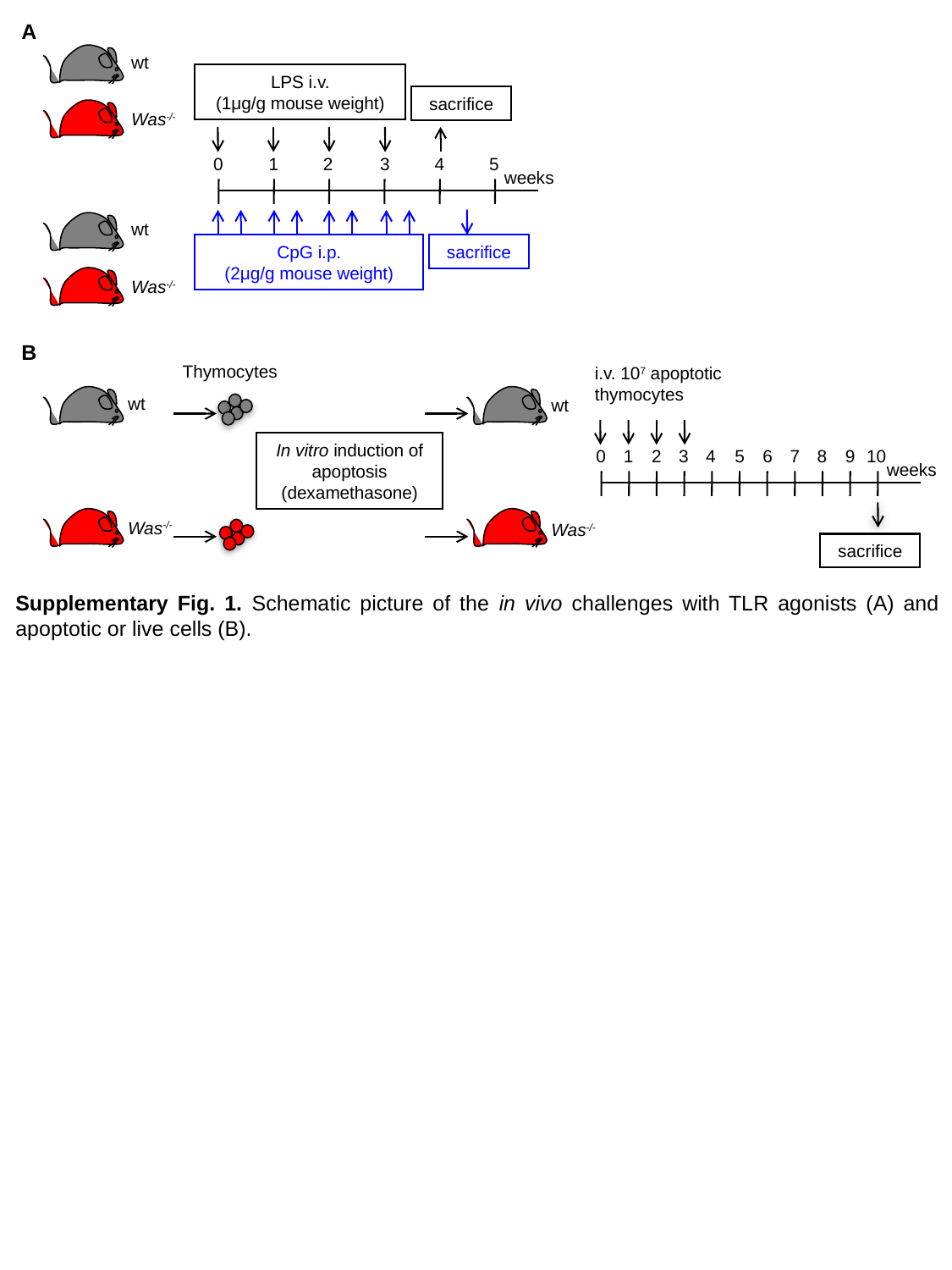

A
wt
LPS i.v.
(1μg/g mouse weight)
sacrifice
Was-/-
0
1
2
3
4
5
weeks
wt
CpG i.p.
(2μg/g mouse weight)
sacrifice
Was-/-
B
Thymocytes
i.v. 107 apoptotic thymocytes
wt
wt
0
1
2
3
4
5
6
7
8
9
10
weeks
sacrifice
In vitro induction of apoptosis (dexamethasone)
Was-/-
Was-/-
Supplementary Fig. 1. Schematic picture of the in vivo challenges with TLR agonists (A) and apoptotic or live cells (B).

## Slide 2
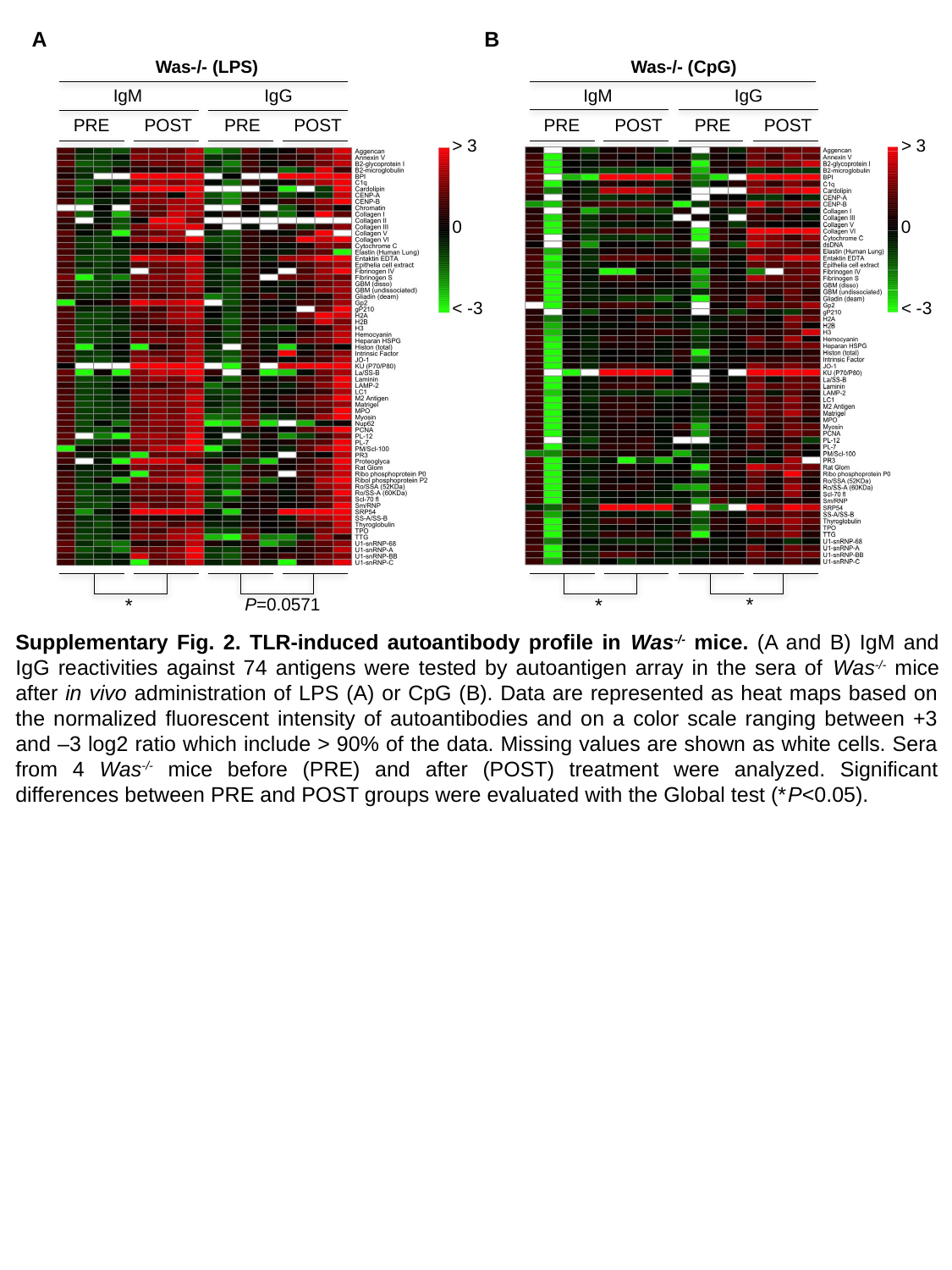

A
B
Was-/- (LPS)
IgM
IgG
PRE
POST
PRE
POST
> 3
0
< -3
*
P=0.0571
Was-/- (CpG)
IgM
IgG
PRE
POST
PRE
POST
> 3
0
< -3
 *
*
Supplementary Fig. 2. TLR-induced autoantibody profile in Was-/- mice. (A and B) IgM and IgG reactivities against 74 antigens were tested by autoantigen array in the sera of Was-/- mice after in vivo administration of LPS (A) or CpG (B). Data are represented as heat maps based on the normalized fluorescent intensity of autoantibodies and on a color scale ranging between +3 and –3 log2 ratio which include > 90% of the data. Missing values are shown as white cells. Sera from 4 Was-/- mice before (PRE) and after (POST) treatment were analyzed. Significant differences between PRE and POST groups were evaluated with the Global test (*P<0.05).

## Slide 3
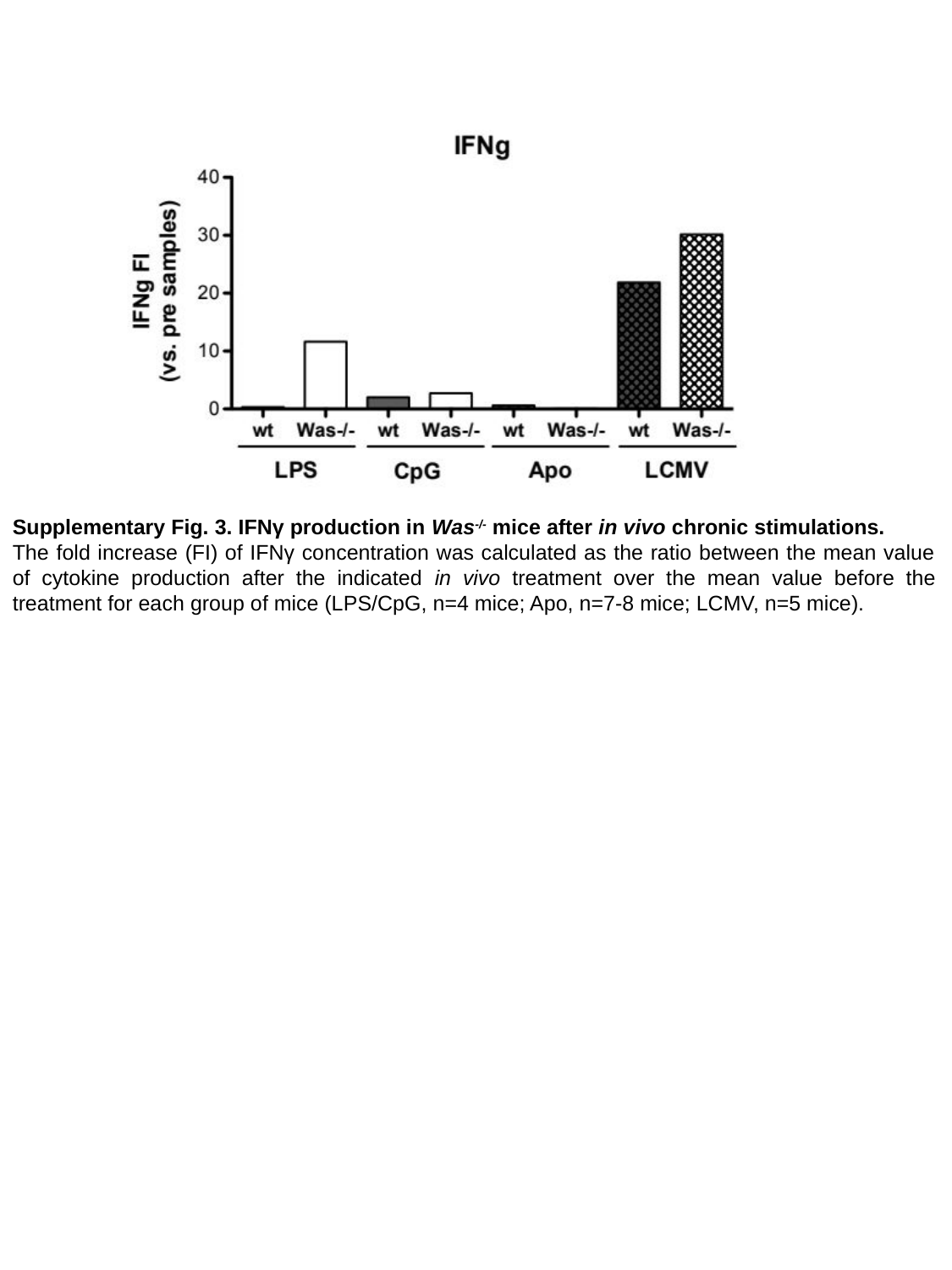

Supplementary Fig. 3. IFNγ production in Was-/- mice after in vivo chronic stimulations.
The fold increase (FI) of IFNγ concentration was calculated as the ratio between the mean value of cytokine production after the indicated in vivo treatment over the mean value before the treatment for each group of mice (LPS/CpG, n=4 mice; Apo, n=7-8 mice; LCMV, n=5 mice).
